# The converging burdens of infectious and non-communicable diseases in rural-to-urban migrant Sub-Saharan African populations: a focus on HIV/AIDS, tuberculosis and cardio-metabolic diseases

**DOI:** 10.1186/s40794-015-0007-4

**Published:** 2015-08-14

**Authors:** Nasheeta Peer

**Affiliations:** 1 Non-communicable Diseases Research Unit, South African Medical Research Council, 491 Ridge Road, Overport, Durban, 4001 South Africa; 2 Department of Medicine, University of Cape Town, Cape Town, South Africa

**Keywords:** Infectious diseases, HIV/AIDS, Tuberculosis, Non-communicable diseases, Cardiovascular disease, Hypertension, Diabetes, Migrant, Co-infection, Africa

## Abstract

Africa has the unenviable challenge of dealing with a double burden of disease: infectious diseases (IDs) such as HIV/AIDS and tuberculosis are high while non-communicable diseases (NCDs) are rapidly rising in the region. Populations with increased susceptibility to both include migrants. This review highlights the susceptibility of rural-to-urban migrants in Sub-Saharan Africa to the IDs of HIV/AIDS and tuberculosis, and to NCDs, particularly cardiovascular diseases. The disruption that occurs with migration is often accompanied by unhealthy exposures and environments. These include partaking in risky sexual practices and a subsequent greater risk for HIV infection in migrants than the general populations which contributes to the spread of the disease. Migrants frequently work and live in conditions that are poorly ventilated and overcrowded with suboptimal sanitation which increases their risk for tuberculosis. Considering that migrants have an increased risk of acquiring both HIV/AIDS and tuberculosis, and in view of the interaction between these diseases, they are likely to be at high risk for co-infection. They are also likely to facilitate the geographical spread of these infections and serve as conduits of disease dissemination to rural areas. Changes in lifestyle behaviours that accompany migration and urbanisation are exemplified primarily by shifts in physical activity and dietary patterns which promote the development of obesity, diabetes, hypertension and cardiovascular diseases. Urban living and employment is generally less physically exerting than rural routines; when migrants relocate from their rural residence they adapt to their new environment by significantly reducing their physical activity levels. Also, nutritional patterns among migrants in urban centres change rapidly with a shift to diets higher in fat, sugar and salt. Consequently, increases in weight, blood pressure and glucose levels have been reported within a year of migration. Interactions between IDs and NCDs are common; considering that migrants have an increased susceptibility to IDs and demonstrate a rapid rise in their risk for NCDs, the concurrent prevalence of both is likely in this population. There is a need for a combined strategy to combat IDs and NCDs with screening and treatment programmes geared towards this high risk group.

## Introduction: the double burden of disease in Africa

Africa has the unenviable challenge of dealing with a double burden of disease: alongside continuing high rates of infectious diseases (IDs) such as human immunodeficiency virus (HIV)/ acquired immunodeficiency syndrome (AIDS), tuberculosis (TB) and other infections, cardiovascular diseases (CVDs) and diabetes are among the chronic non-communicable diseases (NCDs) rising rapidly in the region [1]. Consequently, there has been a change in the major causes of mortality; while once dominated by IDs, these have shifted to include a combination of IDs and NCDs [2].

Africa has been the epicentre of the HIV/AIDS pandemic; the overall prevalence is about 5-9 % (Table 1) [3] but this exceeds 30 % in 30-39-year-old South African women [4]. Of the estimated 35 million HIV-infected individuals globally in 2014, 71 % (24.7 million) reside in Sub-Saharan Africa (SSA) and the region accounts for 74 % of the worldwide AIDS-related mortality [5]. Closely aligned with the HIV**/**AIDS epidemic is the resurgence of TB, particularly in developing regions. A quarter of the estimated 9 million people with TB in 2013 resided in the Africa region which had the highest rates of cases (280/100 000) and deaths (42/100 000) per capita [6].

**Table 1:** Summary data from reviews or reports on the prevalence of relevant infectious and non-communicable diseases in Sub-Saharan Africa

Source	Disease	Sub-group^a^	Men	Women	Total
Magadi, 2013 [3]	HIV	Poor: urban	-	-	9.2
		Poor: rural	-	-	5.3
		Non-poor: urban	-	-	7.3
		Non-poor: rural	-	-	6.2
WHO [6]	Tuberculosis		-	-	0.03
WHO [10]	Obesity		5.3	11.1	-
Ataklte [8]	Hypertension		-	-	30
Hilawe [13]	Diabetes		5.5	5.9	5.7

^a^Study-specific socio-demographic characteristics

Parallel to this overwhelming burden of IDs is the high prevalence of hypertension, regarded as one of the continent’s greatest health challenges after HIV/AIDS [7]. Hypertension, with an estimated prevalence of 30 % (Table 1) [8], constitutes the foundation for the CVD epidemic in SSA [9]. Associated with hypertension is obesity, an important risk factor for the former as well as for diabetes and CVD. The prevalence of obesity has been steadily increasing in Africa [10] with rates as high as 32 % reported in South Africa [11]. Another key NCD and important contributor to CVD is type 2 diabetes which was low in Africa at about 5-6 % [12, 13]. However, the number of individuals with the disease is expected to rise by 110 % from 19.8 million in 2013 to 41.5 million in 2035 [12].

Although there are numerous other diseases such as malaria and parasitic infections that contribute to the high ID burden in Africa, this review focuses on the IDs of HIV/AIDS and TB. Similarly, NCDs encompass cancers and chronic obstructive airway diseases, with major risk factors including tobacco and alcohol use but the emphasis here is on the development of cardio-metabolic diseases. This review highlights the susceptibility of rural-to-urban migrants in SSA to the IDs of HIV/AIDS and TB, and to NCDs, particularly CVDs. English language studies on migrants, HIV/AIDS, TB and CVD risk factors such as hypertension and diabetes in SSA were obtained from PubMed. The reference lists of articles identified were also examined for relevant studies. Prevalence data were obtained from good quality systematic reviews and World Health Organisation reports.

## Review

### Migration: a rapidly rising phenomenon

Migration today takes many forms including international and national mobility with urban-to-urban relocations or rural-to-urban movements; the latter is the focus in this review. These relocations may be permanent or circular to urban areas or seasonal (short-term) mobilisations within or between urban and rural areas. There has been a rise in circular migration in Africa because of cheaper transport, rural attachments and poor urban living conditions [14].

Populations at high risk of both IDs and NCDs include migrants, particularly those involved in rural-to-urban migration [15]. From time immemorial man has migrated in search of better opportunities. This phenomenon continues today on a global and ever increasing scale with ongoing migration of people in search of gainful employment, hopes for a better life and an escape from poverty.

This is especially true for Africa where the majority of the population eke out a subsistence living. Migration in the region is occurring at unprecedented rates with subsequent rapid increases in urban populations; urbanisation in the region is currently occurring faster than on any other continent [16]. Growth in urban populations in the region is currently averaging 4.5 % per annum with the 2020 urban population expected to reach 487 million [17].

Although young men traditionally form the bulk of migrants in Africa, the independent movement of women has increased over the last few decades [18]. Of the women who are increasingly migrating about 50 % of them are also employed [19]. There is a demand in urban areas for housemaids and nannies, and for workers in the euphemistically termed ‘entertainment industry’ [18].

## Migration: a vector for disease dissemination

Considering the rationale for migration, it is ironic that the disruption that occurs with migration is often accompanied by unhealthy exposures and environments, and the stress associated with the relocation may also have health consequences [19]. Although migration may translate into economic benefits for the individual and their families the economic advantage conferred by migration is often counterbalanced by the toll exacted in terms of ill health.

### HIV/AIDS

In recent times with technological advances and greater global connectedness, and the increased volume of swifter and more efficient transport systems in today’s mobile world, opportunities for disease spread have increased exponentially [20]. A prime example is the rapid spread of the HIV infection over a short time span via corridors of population movement between urban settings, and from urban to rural regions [21]. The disease has spared no corner of the globe spreading rapidly through high levels of mobility to become a global epidemic in only two decades [20].

In Africa, initially healthy migrants such as miners living away from home serve to hasten the transmission of IDs, once they are infected, by creating a bridge between populations. Geographical areas are connected through the mobility of infected individuals and disease spread usually from higher (destination areas) to lower (places of origin) prevalence areas [22, 23]. Thus, in today’s highly interconnected world the movement of people is a key factor influencing the spread of disease with migration becoming an important determinant of global health [22].

Numerous factors lead to migrants initiating and partaking in risky sexual practices, the primary route for the spread of HIV infection, in their urban destinations. Migration may encourage risky sexual behaviours through the disruption of family life and normal sexual relations when separated from their families and spouses for prolonged intervals [22–24]. Migrants are likely to experience feelings of isolation coupled with loneliness when living away from home [23, 24]. Also, there may be fewer social constraints on sexual behaviour in urban centres compared with rural areas which may lead to weakening of social inhibitors [22, 25]. Changing socio-cultural norms with greater freedom afforded and the anonymity associated with living in cities and the nature of work are other factors likely to promote risky sexual behaviours [23]. Furthermore, many migrants tend to have low levels of literacy and skills resulting in a lack of knowledge about and low utilisation of available HIV prevention services at their destination abode [23].

Male migrants separated from their partners often find substitutes, which may include potentially riskier partners such as commercial sex workers. Migrant men may thus have a greater likelihood of engaging in risky behaviours than their non-migrant counterparts. They may also tend to move to or through areas with relatively higher prevalence of HIV infection [25]. Thus, initially healthy migrant men, through their increased exposure to HIV in high risk urban settings, acquire the infection.

Numerous studies in Africa have demonstrated the close relationship between mobility and the spread of HIV infection. Migrants, compared to non-migrants, in Ghana, Kenya and Malawi reported more sexual partners and less frequent condom use [25]. Moreover, in Senegal, internal seasonal migration was the only sociological or behavioural factor linked to the spread of the virus in a rural area within the country [20]. In accordance with these findings, Ugandan and South African migrants were 3-times more likely to be infected with HIV than non-migrants [26]. Similarly, in urban Cameroon, HIV prevalence was found to be 5-times greater in mobile compared to resident men; mobile men reported more risky sexual behaviours in terms of an increased number of partners and one-off contacts [20]. In Kenya, where rural-to-urban migrants contribute substantially to slum settlements [24], the HIV prevalence was more than twofold higher in the latter than in non-slum urban residents [27]. Further highlighting the plight of the urban poor was their much higher prevalence of HIV infection compared to their rural counterparts (Table 1). In contrast, HIV prevalence was similar among non-poor urban and rural residents, thereby emphasising the susceptibility of poor migrant populations to the disease [3]. Migrants are thus at greater risk for HIV infection than the general populations and contribute to the spread of the disease [23].

It is not only migrant men who engage in risky sexual behaviour but also their female counterparts. Female migrants have also been found to have more sexual partners than female non-migrants [21, 25]. A South African study found that female migrants compared to non-migrants had 48 % greater likelihood of being HIV-infected [21]. Noteworthy is that the overall higher odds of being HIV-infected in male and female migrants combined was 27 % compared to their non-migrating counterparts. This illustrates that the costs of migration for HIV risk are particularly disadvantageous to women. Higher risk behaviour linked to migration was associated with a greater likelihood of acquiring HIV infection in women than in men. Furthermore, the number of sexual partners increased the odds of HIV infection in female migrants to a greater extent than for male migrants [21].

Notably, migration has important implications not only for the migrant him/herself and for the destinations that host the migrants but also for those who are left behind [22]. The absence of a partner at home for an extended period may influence the remaining partner’s sexual behaviour because of a combination of sexual and financial needs [14]. The risk of extramarital relationships is higher as well as the spread of HIV infection if accompanied by unprotected sex [14]. The stay-at-home wives of migrants may be more likely to engage in extramarital relationships than those of non-migrant men [25]. This emphasises that migration affects the sexual behaviour of not only both genders but also of both the migrants and their stay-at-home partners and highlights the complexities associated with the spread of HIV infection.

### TB

Frequently, migrant working and living conditions are poor with informal living conditions, a lack of adequate housing, poor ventilation, overcrowding and poor access to safe water and sanitation [15, 22]. These environments are highly conducive for disease transmission exposing migrants to greater risks of infection. Also, by living in areas with higher disease prevalence, their exposure to infected individuals is increased. A prime example is the spread of TB in southern Africa to and through migrant mine workers [22]. These migrant workers had a significantly higher risk exposure at the workplace because of unclean working conditions, exposure to silica dust, overcrowded housing and inadequate sanitation.

Furthermore, they unwittingly transferred the disease from high prevalence urban hotspots to rural areas; on their annual visits home to their original residences, migrants served to disseminate these diseases to rural populations. Their partners, families and community members were exposed to a disease that, until then, had not been detected in rural regions [22]. This underscores the critical role of human migration as a vector for disease dissemination, particularly in the setting of high exposure in urban areas [19, 22].

### Circular migration

Many migrants are now able to frequently return home because of improved transportation between rural and urban areas and on account of greater flexibility of work contracts [28]. Therefore, in a highly mobile world, the continued circulation of migrants between places of origin and destination may accelerate the spread of disease [19]. The impact on the spread of TB is evident with more frequent home visits by migrants rendering rural families more vulnerable to potential infection. Additionally, migrants in poor health will more likely spend longer time at home, thereby exposing more people in rural communities to the disease [22].

With regards to the spread of HIV infection, the influence of frequent circular migration may be more nuanced. It is possible that an increased frequency of return may potentially be protective if this deters migrants from taking additional sexual partners while away [22]. Nonetheless, if already infected, the frequently returning migrants may increase the risk of HIV transmission with more frequent exposures of the uninfected partners. Little is known though about the impact of frequently returning home migrants on HIV transmission and further research is required to explore this behavioural pattern [22].

## Migration: influences on the uptake of lifestyle risk factors

It is not only the spread of IDs that is influenced by rural-to-urban migration; there is an equally significant influence on lifestyle behaviours that contribute to the development of NCDs, particularly diabetes and CVDs. Changes in behavioural patterns that accompany migration and urbanisation are exemplified primarily by shifts in physical activity and dietary patterns which promote the development of obesity, an important risk factor for diabetes, hypertension and CVD.

### Physical activity

Behavioural modifications in migrant populations are influenced by the physical, economic and social environments in which individuals reside today and have evolved rapidly in recent times [29]. The changes, which are occurring predominantly in urban settings, include revolutions in transportation, communication, workplace and domestic-entertainment technologies. Consequently, urban living and employment is generally less physically exerting than rural routines. Modes of work and transportation, the main contributors to physical activity in Africa, differ vastly between urban and rural areas. Urban compared to rural living is usually associated with better road networks and easier access to public transport, which translates into less time spent walking to a particular destination. Also, technology and economic incentives tend to discourage activity; technology by decreasing the energy requirements for routine daily activities, and economics by greater reimbursements for sedentary than active work [30].

Therefore, when migrant populations relocate from their rural residence they are likely to significantly reduce their physical activity levels because of adaptations to urban living and employment. Unwin and colleagues [31] reported the rapid uptake of urban patterns of lifestyle behaviours in rural-to-urban migrants in Tanzania. They found a 53 % and 22 % reduction in vigorous physical activity levels in just a year in these migrant men and women, respectively. This indicates that even in many parts of Africa physical activity patterns over the past few decades have shifted from labour-intensive lifestyles to more sedentary and less physically demanding activities [32]. Reduced physical activity is likely a key contributor to rising metabolic diseases in the region [33], particularly among the urban poor who have reduced access to exercise facilities such as gyms, parks, *etc.*

### Diet

In addition to lower physical activity levels linked to urban compared to rural living, there are marked differences in dietary food patterns in these regions. Rural diets in Africa tend to comprise traditional plant based diets high in carbohydrates and fibre and low in fat. In contrast, urban diets are usually higher in fats, sugar and salt, and lower in carbohydrates, vitamins, trace elements and fibre intake [34].

Nutritional patterns among migrant populations living in urban areas are likely to shift to diets that predispose to the development of atherosclerosis. Saturated fat intake was significantly higher in Tanzanian male migrants a year after relocating from rural to urban areas [31]. Among the female migrants, while the rise in saturated fat intake was not significant, consumption of fried potatoes, red meat and soft drinks increased. These data underline the rapid nutritional transition that occurs in rural-to-urban migrants over a short period of time. It also emphasises that improvements in socioeconomic status that may accompany migration to urban centres do not necessarily translate into improved nutritional status. Notably, the uptake of urban diets in South African migrants, including the intake of fast foods and foods high in sugar and fats, was often perceived as progress from rural diets [35].

Interestingly, Unwin and colleagues reported an increase in the consumption of fruits and vegetables in Tanzanian migrants [31]. Likewise, in South Africa, the intake of fruits and vegetables was higher in urbanised professionals than in farm workers and other rural residents [36]. This indicates that urban compared to rural living may offer an advantage in terms of access to a more varied diet [31] as well as perhaps greater affordability related to increased disposable income.

### Overweight and obesity

The combination of reduced physical activity levels with high energy diets in migrant populations quickly leads to weight gain [31] with some even considering this an achievement [35]. Rapid increases in weight, waist circumference and body mass index (BMI) were found in both male and female migrants in Tanzania [31]. These measurements were all significantly higher at one year compared to baseline with apparent increases in BMI reported from about four months after migration. In a study from Cameroon, Sobngwi and colleagues observed that individuals who had migrated from rural to urban areas within the previous 2 years had higher mean BMI compared to rural residents [37].

In accordance with the close relationship between adiposity and diabetes, the latter study found fasting blood glucose levels to be higher in migrants compared to rural residents. The continuous migration to urban centres will likely lead to increases in obesity prevalence and a subsequent rise in diabetes among migrant populations.

### Blood pressure

Consistent with the pathophysiology of hypertension, blood pressures (BPs) are known to increase more quickly over time following the development of obesity, change in dietary habits and stress [37]. Accordingly, studies in Africa have shown a rise in BP in migrant populations. Increases in BP in the Kenyan Luo migration study were observed as early as within 3–24 months of rural to urban migration [38]. Also, urban migrants in Cameroon had higher BP compared to rural residents. Ongoing migration is therefore likely to escalate the increasing and high burden of hypertension on the continent, especially among the poor who may have a greater exposure to stress due to economic difficulties.

## Interaction between IDs: TB and HIV

The advent of the HIV/AIDS pandemic has witnessed a dramatic shift in the delicate equilibrium between effective host immunity and florid TB infection with life-threatening consequences [39]. Individuals with latent TB infection, which comprises about a third of the population, have a vastly greater risk of developing active TB if immunocompromised by concurrent HIV infection [39, 40]. Therefore, despite the multifactorial aetiology of TB, HIV infection is the key driver of the epidemic today with 13 % of TB cases co-infected with HIV worldwide [39].

The burden is particularly high in Africa where there has been an exponential rise in overt TB in the immunocompromised. Countries in eastern and southern Africa that are most affected by HIV have witnessed a fivefold or more rise in their TB caseload. Incidence rates in these regions are now comparable with those found in Europe in the 1950s before the appearance of anti-TB drugs [40]. Compared to the global TB incidence of 16/100 000 among the HIV-infected, the rate in Africa is 94/100 000 [6]. Notably, co-infection leads to worse outcomes and is responsible for a high proportion of TB-related mortality [39].

Considering that migrant populations have an increased risk of acquiring both HIV/AIDS and TB, and in view of the interaction between these diseases, they are likely to be at high risk for co-infection. Moreover, migrants are usually among the poorest and most vulnerable in a community; this coupled with their inadequate access to healthcare services, probably attributed to their new surroundings, may likely contribute to a delay in diagnosis and optimal treatment. There is thus an important need to empower migrant communities through increased social awareness and integration, and frequent health communications [41].

## Interaction between IDs and NCDs

There is an expanding body of literature that demonstrates a convergence of IDs and NCDs with these conditions displaying synergism whereby the one exacerbates or influences the development of the other [2]. That two of the most common IDs seen in Africa, TB and HIV/AIDS, not only co-exist but interact with NCDs is of great concern [1, 17, 42]. This is particularly worrisome in migratory populations who not only have an increased susceptibility to IDs but also demonstrate a rapid rise in their risk for NCDs. The co-occurrence of IDs and NCDs is thus likely to be prevalent in migrant populations. However, there is scant data in Africa available on this topic.

### HIV and NCDs

A key contributor to the changing pattern of mortality in Africa has been the widespread introduction of anti-retroviral therapy (ART) which has seen the lifespans of HIV-infected individuals prolonged and the infection now considered a chronic condition [19]. Consequently, with greater longevity, HIV-infected individuals face the same risk for acquiring CVDs and other NCDs as HIV-uninfected populations *i.e.* exposure to traditional risk factors for cardio-metabolic diseases.

Moreover, the presence of HIV-infection itself may contribute, both directly through immune activation and inflammation, and indirectly through immunodeficiency, to metabolic and inflammatory changes and thereby further raise the risk for CVD [43, 44]. HIV-infection per se promotes endothelial dysfunction, atherosclerosis and thrombosis.

Additionally, the receipt of combination ART especially those that include protease inhibitors, while revolutionising the care of HIV-infected individuals, has been associated with frequent and sometimes severe metabolic side effects. ART causes insulin resistance and decreased insulin secretion [43] thereby increasing the risk for diabetes and the metabolic syndrome [42], worsens lipid profiles [1, 17, 42], in particular low high-density lipoprotein cholesterol levels, and increases the risk of developing CVD [17]. The increased cardiovascular risk of some ART may be through their impact on metabolic and body fat parameters, but also possibly through other factors that are currently unclear [44].

### TB and NCDs

The relationship between TB and diabetes, a key NCD, has long been recognised but underappreciated with these two conditions influencing one another by multiple pathways [45]. The presence of diabetes probably impairs the immune responses needed to control bacterial infections [46, 47], thereby increasing susceptibility to TB. Diabetes contributes substantially to incident TB globally: a systematic review of cohort studies found that individuals with, compared to without, diabetes have a threefold greater risk of developing active TB infection [46]. In Uganda, 8.5 % of patients admitted with TB were diagnosed with diabetes [48]. This was higher than the 2.2 % diabetes prevalence in the general Ugandan population or the 6.4 % prevalence in the hospital medical units during the study period.

Infection with TB in individuals with diabetes is often associated with poor glycaemic control [47, 48]. The increased pro-inflammatory state that accompanies TB where counter-regulatory stress hormones such as epinephrine, cortisol and glucagon are released is likely to induce reactionary hyperglycaemia [48]. Additionally, anti-TB drugs such as rifampicin may induce hyperglycaemia directly or indirectly via interactions with oral hypoglycaemic agents while pyrazinamide can disrupt glycaemic control [45, 47].

Moreover, co-morbid presentation of diabetes with TB can worsen treatment outcomes and exacerbate the severity of TB infection, particularly if diabetes is poorly controlled [1, 17, 45, 47]. There is a greater risk for TB treatment failure and death in individuals with diabetes [47]. Migrant population may likely succumb to the worse case scenarios accompanying co-infection because of their frequent difficulties in accessing healthcare services. This may lead to delayed treatment and an absence of on-going and regular attendance at healthcare facilities.

Furthermore, seeing that migrant populations are particularly vulnerable to acquiring TB infection, and in conjunction with their rapid uptake of urban lifestyles, they may be at higher risk for co-infection with TB and diabetes. In depth research is needed to investigate the pattern of this co-infection in migrant African populations.

## Conclusion

The greater susceptibility for HIV and TB infections among migrant populations in Africa and their rapid uptake of risk factors that predispose to diabetes and CVDs highlight their vulnerability to the dual burdens of IDs and chronic NCDs. This is related to their high risk sexual behaviour, their uptake of unhealthy lifestyles as well as their suboptimal working and living conditions. That migrants in Africa are generally among the poorest populations in their places of destination and likely to be amongst the most vulnerable to these diseases accords with the strong social gradient that frequently correlates with both IDs and NCDs [49].

There is a need for a combined strategy to combat these diseases with screening and treatment programmes geared towards this high risk group with overlapping care needs. Although more research is needed on an optimal management approach for the dual disease burden in migrant populations, there are measures that can be introduced to improve detection. These include screening for TB and HIV infection in high risk areas where migrants are known to congregate such as in slums. Simultaneous targeted screening for diabetes with lower clinical thresholds among new TB cases or those at high risk need to be implemented [15]. Noteworthy is that the cost of adding NCD screening to community-based HIV testing programmes have been shown to be relatively low [50]. The opportunity to leverage the ID healthcare infrastructure for integrated disease management underscores the need for the healthcare team to be skilled in the screening and treatment of both IDs and NCDs. To curb the tide of these conditions, the integration of their management is imperative, especially because of the increased susceptibility to IDs or NCDs if one is present.

Moreover, the integration of public health activities for IDs and NCDs, particularly in vulnerable groups, should extend beyond healthcare services to encompass prevention, a crucial component for successful management of both burdens [15]. It has been suggested that, owing to the high mobility of migrants, intervention measures that target this population should be implemented in rural areas prior to their departure [26]. Such programmes need to promote condom use and advice on safe sex, awareness of inadequate living conditions that facilitate the spread of TB, and the importance of physical activity and optimal diets.

## Abbreviations

AIDS: Acquired immunodeficiency syndrome
ART: Anti-retroviral therapy
BP: Blood pressure
BMI: Body mass index
CVD: Cardiovascular disease
ID: Infectious disease
HIV: Human immunodeficiency virus
NCD: Non-communicable disease
SSA: Sub-Saharan Africa
TB: Tuberculosis
